# Innovative Application of Salophen Derivatives in Organic Electronics as a Composite Film with a Poly(3,4-Ethylenedioxythiophene)-poly(styrenesulfonate) Matrix

**DOI:** 10.3390/polym16182622

**Published:** 2024-09-17

**Authors:** María Elena Sánchez Vergara, Omar Jimenez Correa, Ricardo Ballinas-Indilí, Ismael Cosme, José Ramón Álvarez Bada, Cecilio Álvarez-Toledano

**Affiliations:** 1 Faculty of Engineering, Universidad Anáhuac México, Av. Universidad Anáhuac 46, Col. Lomas Anáhuac, Huixquilucan 52786, State of Mexico, Mexico; omar.jimenez@anahuac.mx (O.J.C.); ramon.alvarez@anahuac.mx (J.R.Á.B.); 2 Department of Chemical Sciences, Facultad de Estudios Superiores Cuautitlán Campo 1, Universidad Nacional Autónoma de Mexico, Avenida 1o de Mayo s/n, Colonia Santa María las Torres, Cuautitlán Izcalli 54740, State of Mexico, Mexico; ricardoballinas1989@gmail.com; 3 National Institute of Astrophysics, Optics and Electronics (INAOE), Luis Enrique Erro #1, Tonantzintla 72840, Puebla, Mexico; ismaelcb@inaoep.mx; 4 Chemistry Institute, Universidad Nacional Autónoma de México, Circuito Exterior s/n, Ciudad Universitaria, Mexico City 04510, Mexico; cecilio@unam.mx

**Keywords:** PEDOT:PSS, salophen, composite film, optical properties, electrical behavior

## Abstract

In this work, we present the innovative synthesis of salophen (acetaminosalol) derivatives in a solvent-free environment by high-speed ball milling, using a non-conventional activation method, which allowed obtaining compounds in a shorter time and with a better yield. Furthermore, for the first time, the salophen derivatives were deposited as composite films, using a matrix of poly 3,4-ethylene dioxythiophene:polystyrene sulfonate (PEDOT:PSS) polymer. Significant findings include the transformation from the benzoid to the quinoid form of PEDOT post-IPA treatment, as evidenced by Raman spectroscopy. SEM analysis revealed the formation of homogeneous films, and AFM provided insights into the changes in surface roughness and morphology post-IPA treatment, which may be crucial for understanding potential applications in electronics. The optical bandgap ranges between 2.86 and 3.2 eV for PEDOT:PSS-salophen films, placing them as organic semiconductors. The electrical behavior of the PEDOT:PSS-salophen films undergoes a transformation with the increase in voltage, from ohmic to space charge-limited conduction, and subsequently to constant current, with a maximum of 20 mA. These results suggest the possible use of composite films in organic electronics.

## 1. Introduction

**Figure 1:**
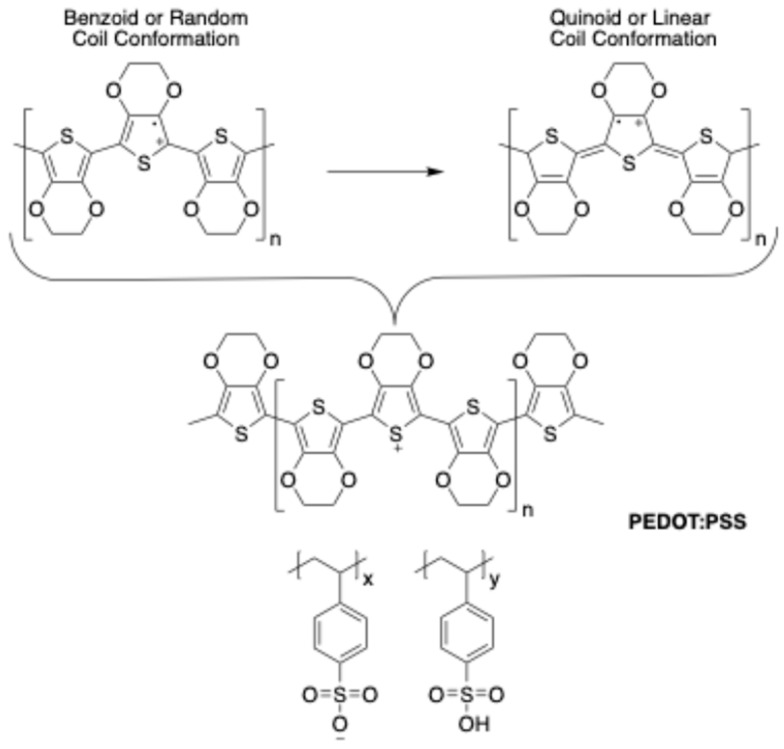
Chemical structure of PEDOT:PSS and the benzoid and quinoid forms of PEDOT.

Poly(3,4-ethylenedioxythiophene) (PEDOT) is a highly conductive and transparent polymer that can be dispersed in water by using poly(styrenesulfonate) (PSS) as a counter ion. This leads to poly 3,4-ethylene dioxythiophene:polystyrene sulfonate (PEDOT:PSS) (Figure 1), which is a polymer that has been widely utilized as a transparent electrode material due to its outstanding properties of high optical transparency in the visible range, high mechanical stability, and high electrical conductivity [1,2]. It is lightweight, easy to process and deposit by spin coating and spray coating, and ink-jet-printable. PEDOT:PSS has a work function of between 4.5 eV and 5.2 eV, roughly comparable with the work function of copper (4.7 eV), which is favorable for applications in hybrid crystalline silicon solar cells [1]. The polymer PEDOT:PSS has also been extensively considered for constructing a variety of organic electronic devices, such as transistors, thermoelectric devices [2], and sensors, due to its low cost, toughness, easy processing, and high conductivity [3]. Because of its multiple applications, PEDOT:PSS is one of the most attractive and commercialized conducting polymers [4]. For instance, in the case of electroconductive hydrogels, a PEDOT:PSS solution was added to a gelatin solution [5]. To boost the performance of inverted perovskite solar cells, a composite-hole transporting layer of PEDOT:PSS/graphene quantum dots has been used [6]. By incorporating the conducting polymer, PEDOT:PSS, into silk fibroin (SF), it developed matrices for human corneal epithelial cell and innervation support [7]. A highly stretchable on-skin strain sensor based on a conductive carboxymethyl cellulose (CMC)/PEDOT:PSS composite film has also been developed [8]. Furthermore, it is possible to significantly increase the sensitivity of ferroelectric polymer P(VDFTrFE)-based mechanical/thermal sensors when the conductive polymer PEDOT:PSS works as an electrode [9]. Finally, PEDOT:PSS has been used to provide a new method to fabricate intelligent fire-warning temperature sensors for high-temperature operation, ensuring fire safety and human movement guidance [10].

Another interesting aspect of this polymer is that the PEDOT portion may be present in two different forms, namely benzoid and quinoid (see Figure 1). The benzoid structure may be the favored structure in the random coil conformation, while the quinoid structure is favored in the linear or expanded coil conformation. In a linear conformation, the interactions among PEDOT:PSS chains are expected to be stronger than those among PEDOT:PSS chains in a coil conformation [11]. That is because benzoid structures have two C=C covalent bonds in the coil-shaped thiophene, while the quinoid structure has one C=C covalent bond in the thiophene having the linear, expanded coil shape. In the 1400–1500 cm^−1^ range band of the PEDOT Raman spectrum, the Raman frequency is related to the C=C covalent bond vibration. Thus, the chains within the PEDOT molecular structure can change from the benzoid to a quinoid structure characterized by a relatively weak bond strength, resulting in the shift of the Raman spectrum to the left. Consequently, as the structure of the PEDOT polymer changes to an expanded (linear) coil shape, the charge-carrier motion between or within the neighboring PEDOT polymer chains and the electrical conductivity are improved [12]. It is hard to find a single application for the benzoid and quinoid forms, although it can be seen, with Raman spectroscopy, that sulfuric acid as a solvent-dopant affects the PEDOT:PSS structure. Solvent doping contributes to conductivity enhancement by changing from benzoid to quinoid the PEDOT resonant structure [13]. A post-treatment with isopropanol is another way to change the benzoid structure to quinoid and thus increase charge transport in solar cells [14]. Another approach to increasing conductivity involves adding ZnO nanoparticles to the PEDOT:PSS system, which has been reported to at least double the conductivity values [15]. Furthermore, conductivity increases have been noticed upon the addition of some organic solvents to a PEDOT:PSS water solution, including glycerol, dimethyl sulfoxide (DMSO), sorbitol, ethylene glycol, and *N, N*-dimethylformamide (DMF) [15]. This is beneficial for use in sensors and flexible electronic devices and could have significant applications in the improvement in electronics in innovative PEDOT-type devices, such as supercapacitors and solar cells [13], biosensors, scaffolds for tissue engineering, electrodes for electrophysiology, and implantable electrodes [16,17].

**Figure 2:**
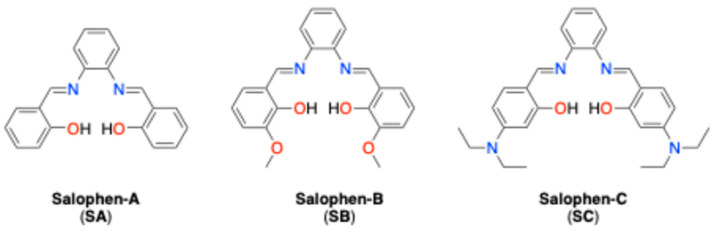
Molecular structure of salophen derivatives.

Due to the significant importance of PEDOT:PSS in molecular electronics, it is considered that its compatibility with other types of organic compounds, such as the ligands generally known as ‘salens’ (*N*,*N*-bis(salicylidene) ethylenediamine) (see Figure 2), should be further studied. This is due to the fact that PEDOT:PSS has poor mechanical properties, which, under service conditions, considerably decreases the lifetime of a device containing films of this polymer. On the other hand, when using it as a matrix in composite films that have particulate reinforcements, an increase in its lifetime would be expected. Given the above, the objective of this work is to report the fabrication and optical and electrical characterization of innovative PEDOT:PSS-salophen composite films, in order to determine their possible use in organic optoelectronic devices. The condensation of salicylaldehyde derivatives with *o*-phenylenediamine leads to the formation of salophen or *N*,*N*’-bis(salicylidene)-*o*-phenylenediamine [18]. The important characteristics of salophen ligands seem to be intrinsically associated to intramolecular hydrogen-bond formation and to the coexistence of several isomeric forms [19]. Schiff bases, with the position of the hydroxyl group in ortho relative to the amino groups, are relevant in this regard due mainly to the O-H^....^N hydrogen bonds leading to three possible tautomers between the keto-amine and enol-imine of the salophen ligands [20,21]. Ligands of the salen- or salophen-type with two nitrogen and two oxygen atoms are relevant for easy synthesis, capability to coordinate with a large number of metal ions, and application potential in many fields [20]. Catalysis is one of the most common applications [20,21,22]; however, these ligands are also used in molecular magnetism [23,24,25], biological activities monitoring [26,27], gas absorption [28,29], light-emitting diodes [30], and field-effect transistors [31]. 

Salen and salophen ligands provide an N_2_O_2_ binding site with a square planar geometry. They can easily coordinate with a wide variety of metal ions of major group elements, and a wide range of metal complexes have been synthesized. The construction of salophen-based materials embedded in covalently bonded polymeric backbones employs different approaches, and the different synthetic strategies are based on the properties of the resulting materials. The different properties of the materials and their potential applications derive from the molecular structure of the salophen unit, the nature of the metal ions, and the dimensionality of their idealized topology [32]. The salophen complexes operate as building blocks to assemble redox-conducting polymers. The transition metal ions have been combined with salophen ligands with various substituents for the tuning of electronic properties of the ligands; metal–salophen polymers seem to possess properties that make them promising candidates for emerging applications in energy conversion and storage [33]. Other applications of metal–salophen complexes include the fabrication of composite-based flexible and sewable electrodes that can be coated on textiles; having good rate capability and long cyclic stability, these materials also have a significant potential in electrode for asymmetric wearable supercapacitor (AWSC) devices [34]. 

On the other hand, mechanochemical ball milling has become one of the most important technologies in chemical synthesis, as it has several advantages in achieving more sustainable and environmentally friendly transformations by offering a greener alternative to traditional synthesis methods, such as solvent-free synthesis; the reaction can be carried out at room temperature without the need for external heating, which substantially reduces energy consumption. In addition, the reaction requires just a simple preparation. A mechanical force is used to drive the chemical reactions, presenting relevant advantages in organic synthesis, such as good scalability, and unusual selectivity with high yields and low reaction times [35]. The synthesis of salophen derivatives has been carried out efficiently while studying the electronic effects of the substituents present in the reactants on the formation of the molecules of interest [36]. It is for the above reasons that the goals of this work are focused on two important aspects. The first is to carry out the synthesis of the salophen derivatives under high-speed ball milling in a solvent-free manner, in which the use of a non-conventional activation method allows to obtain the compounds in a shorter time and with a better yield, according to green chemistry protocols. The second goal is to obtain, for the first time, composite films with salophen derivatives embedded in PEDOT:PSS and to study their optical and electrical properties to determine their potential use in molecular electronics.

## 2. Materials and Methods

The reagents were used as received: *o*-phenylenediamine, salicylaldehyde, 3-methoxysalicylaldehyde, 4-(diethylamino)salicylaldehyde, HPLC ethanol (obtained from a commercial source (Sigma-Aldrich, Carlsbad, CA, USA)), *n*-hexane, and methanol (acquired from Merck-Millipore, Steinheim, NW, Germany). ^1^H and ^13^C NMR spectra were generated in CDCl_3_ with a Bruker 300 Ascend equipment (Bruker, Ettlingen, BW, Germany) operating, respectively, at 300 and 75 MHz. The 0.8% in H_2_O polymeric matrix, poly(3,4-ethylenedioxythiophene):poly(styrenesulfonate) (PEDOT:PSS: [C_8_H_8_O_3_S]_n_-[C_6_H_6_O_2_S]_n_), came from a commercial source (Sigma-Aldrich, St. Louis, MO, USA). 

### 2.1. Synthesis and Characterization of Salophen Compounds

**Scheme 1:**
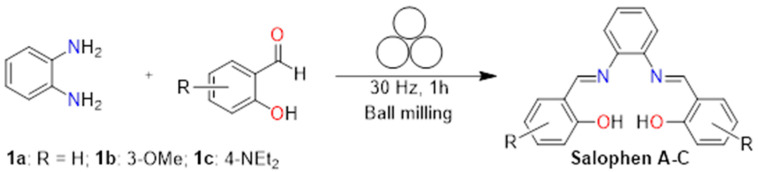
Salophen general reaction **A**–**C**.

The synthesis of salophen derivatives (Scheme 1) was based on a previously reported process [36]. *o*-phenilenediamine (0.1081 g, 1.0 mmol) and 2.0 eq of corresponding aldehyde (**1a**–**c**) were added into a stainless steel jar with a 10 mm stainless-steel ball, and the reaction mixture was milled in the Mini-Mill Pulverisette 23 (Fritsch, Idar-Oberstein, RP, Germany) high-vibratory ball mill, during 1 h without any solvent at a frequency of 30 Hz. The solid product from the jar was washed with hexane and methanol. The mixture was decanted and dried in vacuum at room temperature, and recrystallized from ethanol. It is worth noting that the molecules thus obtained had been previously reported; hence, their physical and spectroscopic data were readily correlated with the available literature information [33,34,35,36,37,38,39]. 

### 2.2. Composite Film Manufacturing

The infrared (IR) spectroscopic analysis was performed in KBr pellets with a Nicolet iS5-FT (Thermo Fisher Scientific, Inc., Waltham, MA, USA) spectrometer. The FTIR range of wavelengths was from 4000 to 400 cm^−1^. In order to obtain the composite films, dispersions of the compounds SA, SB, and SC were prepared in 5 mL of PEDOT:PSS. The dispersion was mixed homogeneously with a Vortex-Genie 2 (Scientific Industries, Bohemia, NY, USA), as well as a 200 Smart Coater (Laurell Technologies Corporation, North Wales, PA, USA), with a spin speed of 950 rpm, spin time of 11.0 s, acceleration of 250 rpm/s, and center time of 9.0 s, used for the manufacture of PEDOT:PSS-salophen compound films. Glass and n- and p-type silicon, as well as fluorine-doped, tin-oxide-coated glass slides (FTO: SnO_2_/F) were used as substrates to obtain composite films fabricated by the spin coating technique. Prior to deposition, the glass and FTO slides were sequentially washed in an ultrasonic bath with methanol, chloroform, and acetone; the silicon slides were washed with hydrogen peroxide and subsequently with methanol and acetone. After depositing the films on the substrates, and to carry out the polymerization of PEDOT:PSS, they were heated to temperatures between 80 and 87 °C. The post-treatment with isopropanol (IPA) was carried out by contacting the solvent vapor with the films, for 5 min, at a temperature of between 40 °C and 45 °C. Raman spectra were measured on the composite films with an NTEGRA Spectra (NT-MDT Inc., Liestal, Switzerland), NT-MDT AFM Raman system using a 532 nm excitation laser (NT-MDT Inc., Liestal, Switzerland). Scanning electron microscopy (SEM) was performed with a FEI Scios DualBeam scanning electron microscope (Thermo Fisher Scientific, Waltham, MA, USA). Atomic force microscopy (AFM) measurements were made with an NTEGRA platform, and the statistical characteristics of the surface were extracted using the Gwyddion 2.66 software. The optical properties, including the transmittance and absorbance on the glass substrate of the composite films, were obtained with a UV–Vis 300 Unicam (Thermo Fisher Scientific, Waltham, MA, USA) spectrophotometer and the program Thermo Insight, in a wavelength range from 190 to 1100 nm. Such properties were also obtained from liquid-based chloroform solutions. A Keithley 4200-5CS-PK1 (Tektronix, Beaverton, OR, USA) auto-ranging picometer with a four-point probe and a circuit for lighting control from Next Robotix (Comercializadora KMox, Mexico City, Mexico) were used to determine, through current–voltage (I-V) measurements, the electrical properties of the glass/FTO/PEDOT:PSS-salophen/Ag devices, using for the anode an FTO-conducting substrate and silver for the cathode. The device was irradiated with an illumination system, using a non-conventional activation method, which provided different types of irradiations: red (1.77 eV), orange (2.0 eV), yellow (2.14 eV), green (2.34 eV), blue (2.64 eV), UV (2.70 eV), and white (peak at 2.57 eV). Also, the I-V behavior of the devices was measured in conditions of darkness and natural light. Finally, external quantum efficiency (EQE) was measured from an AM1.5 solar simulator with a QUESA-1200 system, including a TFSC Instrument (Intercovamex, Cuernavaca, Morelos, México) LED light source under 100 mW/cm white light irradiation.

## 3. Results

**Figure 3:**
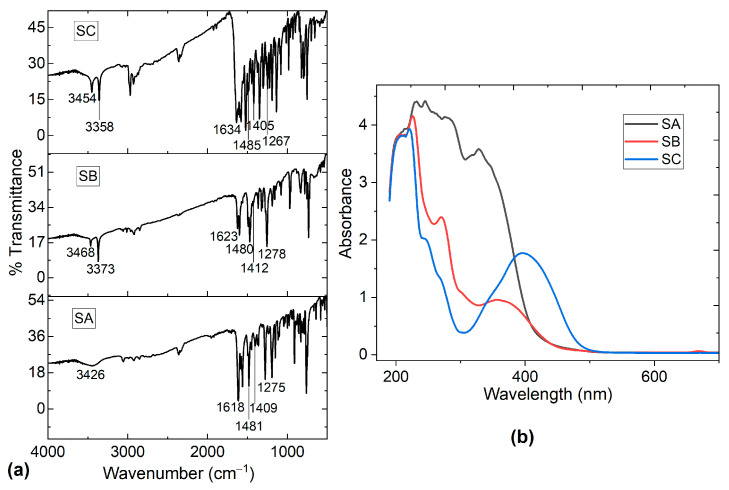
(**a**) FT-IR spectrum of salophen compounds and (**b**) UV-Vis spectrum for SA, SB, and SC in chloroform.

The salophen compounds were obtained by reacting *o*-phenilenediamine with the corresponding salicylaldehyde derivative by a solvent-free methodology. The resulting molecules, as orange solids, were produced in a range yield of 80–90% (from good to excellent). IR and UV-vis spectroscopy and ^1^H-NMR were used to characterize the target molecules. The spectroscopic data were appropriately correlated with those reported in the literature [37,38,39]. The IR spectra in Figure 3a show the characteristic signals to be in the range of 1618–1634 cm^−1^ for C=N stretching vibrations, showing the successful formation of the azomethine structure [36,40,41,42]. A weak absorption band in the range of 2980–2860 cm^−1^ was found for the CH aromatic-stretching vibrations [36]. The C=C stretching vibration of the aromatic rings was assigned to the band around 1481 cm^−1^ [40] and, for C-O stretching, the signal was around 1260–1275 cm^−1^ [40]. The C-C vibration appeared around 1405–1412 cm^−1^, while the spectrum shows a broad band around 3426–3468 cm^−1^, which was assigned to OH [40,41,42]. The UV-Vis absorption spectra of all the salophen compounds was measured in a chloroform solution (10^−4^ M); the absorbance spectra are shown in Figure 3b. According to these absorbance values, optical behavior is observed in salophen compounds, which can be beneficial for their use in organic electronics devices. Differences were observed in the three spectra, related to the structure of each salophen compound. The spectrum of SA showed two bands at 277 and 327 nm assigned, respectively, to π/π* and n/π* intra-ligand transitions [40]; in the spectrum of SB, bands appeared at 270 and 361 nm and, in the spectrum of salophen SC, at 247 and 398 nm. The shift in the spectra was related to an increase in π-conjugation, as well as the addition of an electron donating group (EDG) in the corresponding salophen derivative. Previous IR and UV-vis studies indicate that the synthesis method used was appropriate for the production of salophen compounds.

**Figure 4:**
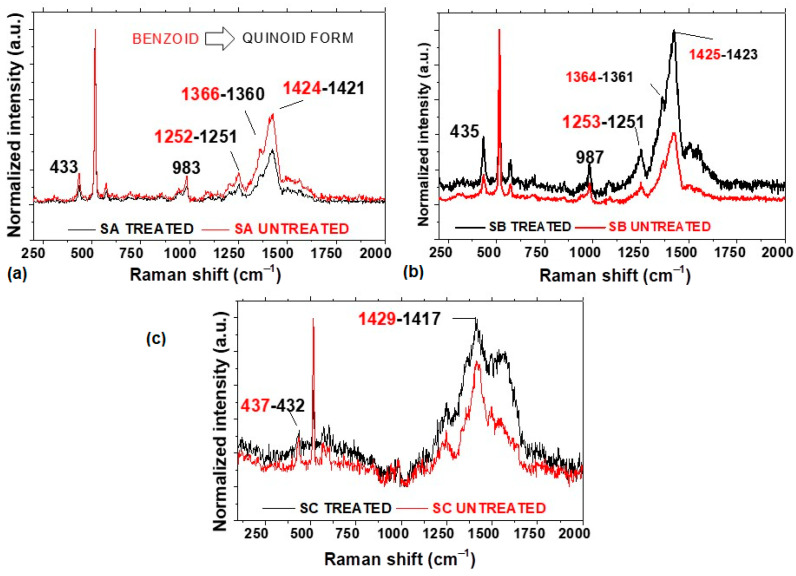
Raman spectroscopy of the (**a**) PEDOT:PSS-SA, (**b**) PEDOT:PSS-SB, and (**c**) PEDOT:PSS-SC composite films.

The thus characterized salophen compounds were embedded in the PEDOT:PSS matrix by means of spin coating, and the deposited composite films were treated with IPA to obtain the quinoid form of PEDOT. Raman spectroscopy is a valuable tool for studying composite films within a PEDOT matrix. Figure 4 shows the Raman spectra of the (a) PEDOT:PSS-SA, (b) PEDOT:PSS-SB, and (c) PEDOT:PSS-SC composite films. In the Raman spectra, it is observed that all samples present the main bands of the PEDOT film. The bands around 434 ± 3, 984 ± 3, and 1250 ± 4 cm⁻¹ are attributed, respectively, to C-O-C deformation, oxyethylene ring deformation, and C_α_-C_α_ inter-ring stretching [43,44]. The band at 1364 ± 6 cm⁻¹ is attributed to the C_β_-C_β_ stretching mode [45,46], and the intense band at 1423 ± 6 cm⁻¹ corresponds to the C_α_=C_β_ symmetrical stretching vibrations in the PEDOT five-membered thiophene ring, which can provide information related to structural changes in the PEDOT chains [43,44,45,46,47,48]. It is important to consider that, in the benzoid form of the C_α_=C_β_ bond, there are two conjugated π-electrons, while, on the C_α_=C_β_ bond in the quinoid form, there are no conjugated π-electrons [43,44,45,46,47,48]. The symmetrical C_α_=C_β_ vibration will exhibit a red shift when there is a change from the benzoid to the quinoid form in the chain structure. In the spectrum shown in Figure 4a for the PEDOT SA composite film, a shift in the signal from 1425 to 1423 cm⁻¹ is observed. Similarly, in the PEDOT SB composite film (Figure 4b), a shift from 1424 to 1421 cm⁻¹ is noted, and finally, in the PEDOT SC film (Figure 4c), a red shift from 1429 to 1417 cm⁻¹ is observed. The shift in the Raman signal observed in the spectra of the PEDOT:PSS films is attributed to changes in the polymer structure and interactions due to post-treatment, leading to a reorganization of the PEDOT nanostructure. This reorganization can alter the conformation of PEDOT chains from a benzoid to a more conductive quinoid structure, affecting the vibrational modes detected by Raman spectroscopy [14]. The quinoid form is dominant in the PEDOT composite films, which may result in an increase in conductivity. Changes in the Raman spectra intensities further confirm the interaction between the particles and the polymer chains [49]. Finally, when comparing the spectra of the films treated with IPA, more defined peaks are observed in the PEDOT:PSS-SA and PEDOT:PSS-SB films. The above is an indication of a higher degree of molecular order relative to these PEDOT films.

**Figure 5:**
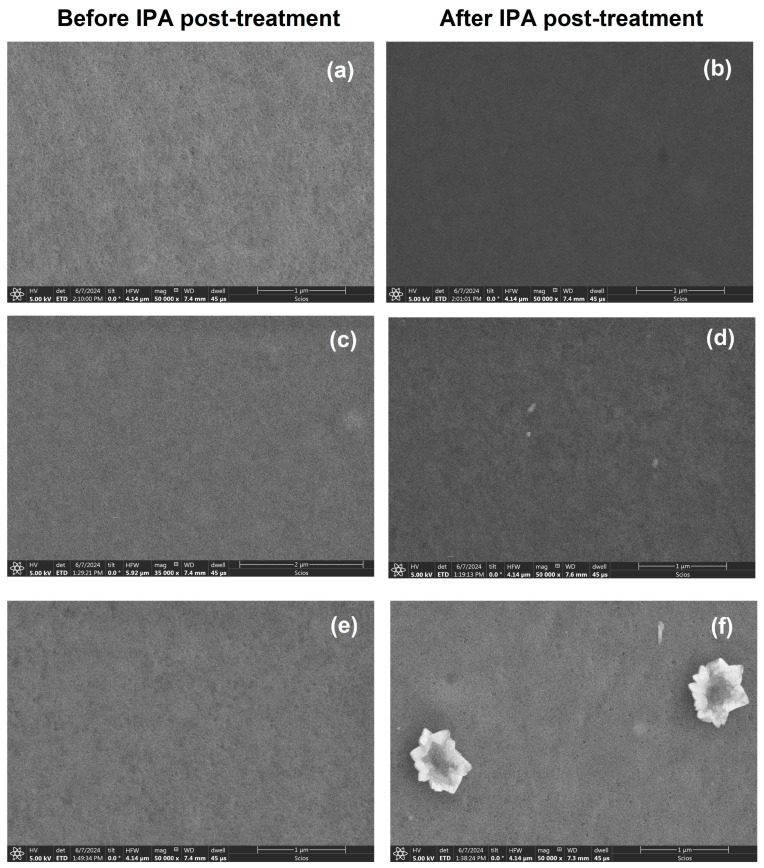
Photomicrographs at 50,000× of the (**a**,**b**) PEDOT:PSS-SA, (**c**,**d**) PEDOT:PSS-SB, and (**e**,**f**) PEDOT:PSS-SC composites films before and after IPA post-treatment.

Scanning electron microscopy (SEM) was performed to analyze film uniformity at the micrometer scale. Figure 5 shows the film micrographs before (Figure 5a,c,e) and after (Figure 5b,d,f) post-treatment with IPA. The SEM images before treatment show homogeneous films with a complete coverage of the substrate. After treatment, the films with SA and SB do not show significant differences. In the case of the film with SC, after post-treatment, flower-shaped structures are observed, likely formed by the desorption of SC particles from the PEDOT matrix. This may leave behind defects in the material. Similarly, the desorption of SC particles may create imperfections on the surface of the PEDOT, leading to structural disorder. This could explain the results obtained by Raman spectroscopy for the PEDOT film, where the increased noise and broader signal widths suggest a disordered structure. This disorder may result from the incomplete embedding of SC particles within the polymer matrix. Further studies are needed to fully understand this mechanism and its impact on the properties of the films. 

**Figure 6:**
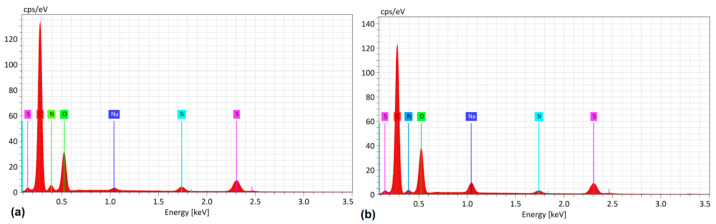
EDS of the PEDOT:PSS-SC composites films (**a**) before and (**b**) after the IPA post-treatment.

Energy-dispersive spectroscopy (EDS) was also performed on the films to verify the presence of the most important chemical elements in the composite films: sulfur from PEDOT:PSS and nitrogen from the salophen compounds. Carbon and oxygen were found in both the polymer matrix and the SA, SB, and SC films (see Figure 6). Since similar results were obtained for all three composite films; only the spectra for the PEDOT-SC films before and after the IPA treatment are presented in Figure 6. In both cases, the salophen elements—S, N, C, and O—were present, along with silicon from the substrate on which the films were analyzed and sodium. To explain the presence of Na, it must be noted that, according to the literature [50,51], the PEDOT positive charge carriers combine with the PSS negative charge carriers through Coulomb interactions to form the PEDOT copolymer. However, the PSS negative charges can also combine with Na ions that may be present in the water of the polymer dispersion (see Section 2). The resulting Na-containing PEDOT exhibits a stronger interaction between the two chains [50]. The above is confirmed by Raman spectroscopy for PEDOT:PSS-SB and PEDOT:PSS-SC, according to Hangyeol Cho et a. [50] and M. de Kok et al. [52]. There was no decrease in intensity in the band at 1423 ± 6 cm⁻¹ due to the presence of Na. However, because of the small amount of Na, there was a transformation from the benzoid form to the quinoid form of the polymer.

**Figure 7:**
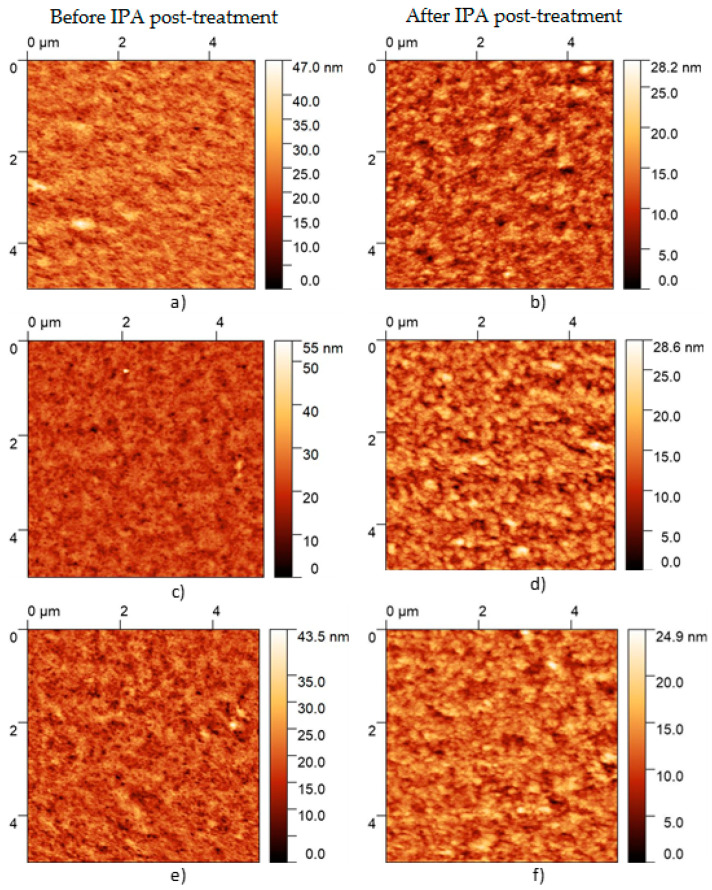
AFM images of the (**a**,**b**) PEDOT:PSS-SA, (**c**,**d**) PEDOT:PSS-SB, and (**e**,**f**) PEDOT:PSS-SC composites films before and after the IPA post-treatment.

AFM was performed with the related objective of complementing the analysis on the film nano-topography. Figure 7 presents the AFM images for the composite films before and after the IPA treatment. The examination of the PEDOT:PSS films before and after the post-treatment reveals notable changes in surface morphology, as indicated by variations in RMS roughness, mean roughness, skewness, and excess kurtosis. The post-treatment in the SA and SC samples led to a marked decrease in RMS roughness, from 3.918 nm to 2.988 nm in SA, and from 3.663 nm to 2.617 nm in SC, and mean roughness, suggesting a smoothing effect on the surface. In contrast, the SB sample showed minimal variation in roughness, with RMS values remaining largely unchanged (from 3.36 nm to 3.40 nm), indicating that the treatment had little impact on the surface texture. The shift in skewness in the SB and SC samples, from negative to positive, points to a redistribution of surface features, with a greater prevalence of peaks post-treatment. Furthermore, the significant reduction in excess kurtosis across all samples suggests a decrease in extreme surface features, leading to a more even surface profile. These observations suggest that the post-treatment process effectively alters the surface characteristics of PEDOT:PSS films, which may have important implications for their functional performance in various applications. For example, in practical multilayer devices, such as solar cells, morphology changes can alter the performance by affecting subsequent layers deposited on top of the films. Defects or irregularities in the morphology of the PEDOT:PSS layer may propagate through the stack, leading to a poor layer adhesion, increased series resistance, or even recombination sites, all of which can degrade the overall device efficiency. RMS roughness values obtained in the PEDOT:PSS films SA to SC are within the average range of those typically observed in thin-film-based devices, demonstrating that the characterized films can be employed in multilayer devices [14].

**Figure 8:**
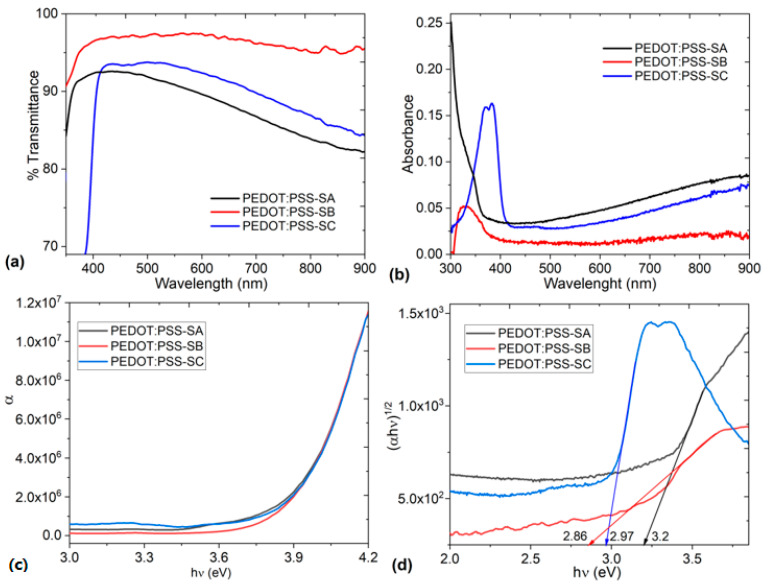
(**a**) Transmittance, (**b**) absorbance, (**c**) absorption coefficient, and (**d**) Tauc plot for the PEDOT:PSS-salophen films.

UV-vis spectroscopy was carried out to study the optical properties of the composite films; the transmittance and absorbance spectra are shown in Figure 8. Regarding transmittance, it is noted that the PEDOT:PSS-SB film has the highest transparency with a maximum value of 97% that is maintained throughout the entire wavelength (λ) range (Figure 8a). The PEDOT:PSS-SA and PEDOT:PSS-SC films, on the other hand, have a similar behavior, resembling that of pristine PEDOT:PSS [53]. PEDOT:PSS-SA has a maximum transparency of 93% and PEDOT:PSS-SC of 94% at λ < 440 nm and λ < 510 nm, respectively, while at a higher λ, the transmittance drops until reaching a minimum close to 80%. These results indicate that the PEDOT:PSS-SB film has the best performance in terms of the percentage of transmittance and its permanence throughout the visible range of the spectrum. The other two films also have high transmittance values in the visible spectrum; so, they could be useful in the manufacture of transparent electrodes for light emission devices, photovoltaic energy, touch screens, or electrochromic devices in the form of smart windows. They could also be used as a hole carrier layer (HTL), since their high transparency plays maximizes the photocurrent intensity in the photoactive layer [54]. Thus, due to the composite films’ transparency, they could be useful as HTL in organic electronics applications. PEDOT:PSS-salophen films are a viable alternative in this regard because of their higher maximum transmittance compared to that of pristine PEDOT:PSS; the maximum transmittance of this polymer is around 85% in the visible range. The presence of salophen favors transparency in the composite films, and the presence and type of substituent in the molecular structure of salophen has a significant effect on the transparency percentage of the composite films. The highest transmittance of 97% in SB is due to the presence of the oxygen and the -OMe electron-donating groups. The small size of the substituent that concentrates the electron-donating capacity is essential to allow the passage of almost constant radiation. On the other hand, the reduction and minimum transmittance of 80% is due to the lack of substituents in SA and the presence of the -NEt_2_ substituents in SC, which, despite having electron-donating capacity, are larger than -OMe.

As expected from the previous results, and according to the spectrum in Figure 8b, the absorbance of the films is very low. Furthermore, this spectrum is also related to PEDOT:PSS, since the initial peaks are observed in the interval between 290 and 400 nm, corresponding to the aromatic rings of PSS [55]. The intensity of the band is lower for the film with SB, suggesting that the quantity of PSS chains in PEDOT:PSS decreases after post-treatment. This would favor stability and conductivity in the composite films [56].

To determine the capacity of the PEDOT:PSS-salophen as semiconductor films, it is necessary to determine their optical band gaps. The band gap in composite films can be obtained by different methodologies, most of them starting from the absorption coefficient α. The α coefficient determines to what extent a particular wavelength can penetrate the PEDOT:PSS-salophen film before being absorbed. The α is obtained from UV-vis spectroscopic measurements and the expression: α = ln(T/d). In this expression, T represents the transmittance, while d = 5.8 nm is the thickness of the film. As can be seen in Figure 8c, α is not constant, but depends on the wavelength of the light being absorbed and specifically on the energy E of the photon. The energy of each photon is inversely proportional to its wavelength λ and can be obtained from E = hν, where h is Planck’s constant and ν is the photon’s frequency. In terms of λ, we have E = (hc)/λ, where c is the speed of light. This means that each photon of shorter λ, such as ultraviolet light, will be more energetic than one of longer λ, such as red light. Figure 8c shows that the curves are very similar to each other and have a sharp edge at α, since light with energy smaller than the band gap is incapable of exciting an electron from the valence band into the conduction band and, therefore, will not be absorbed. The photon absorption probability depends on the probability of the photon interacting in such a way that the electron can pass from one energy band to another. As hν increases, more electrons can interact with the photons, resulting in photon absorption.

The determination of the optical bandgap from Tauc’s method [57] uses the Urbach relation [58] $\alpha h\nu =A{\left(h\nu -E_{g}^{opt}\right)}^{r}$, where the *A* parameter depends on the transition probability, $E_{g}^{opt}$ represents the optical band gap, and the number *r* characterizes the transition process, with *r* = 2 for indirectly allowed transitions typical of amorphous films like PEDOT:PSS-salophen. The graph of ${\left(\alpha h\nu \right)}^{r}$ versus hν was plotted and $E_{g}^{opt}$ was calculated from the determination of the *x*-axis intercept at (αhν)^1/2^ = 0. The resulting values of $E_{g}^{opt}$ are provided in Figure 8d. $E_{g}^{opt}$ has a value of 3.2 eV in the case of the PEDOT:PSS-SA film, 2.86 eV for the PEDOT:PSS-SB film, and 2.97 eV for the PEDOT:PSS-SC film. The change in this value is due to the type of salophen found in each film. SB and SC, with two oxygen and two nitrogen atoms, respectively, in their substituents, are the ones that generate the smallest optical bandgap. This may be due to the presence of the electron-donating groups -OMe and -NEt_2_, which generates a more efficient charge transport than its SA analogue. The best semiconductor behavior is shown by the film with SB, due to its lower $E_{g}^{opt}$. Nevertheless, it should be considered that the SC film has a similar $E_{g}^{opt}$. The film showing a lower semiconductor behavior is the one containing SA. It is worth mentioning that the PEDOT:PSS-SB film, despite having the highest roughness, exhibits the highest transparency and the lowest $E_{g}^{opt}$. Moreover, although the $E_{g}^{opt}$ values obtained in this paper are higher than those for films with inorganic compounds, such as MoO_3_–PEDOT:PSS (1.4 eV) [59], and also higher than for pristine PEDOT:PSS (2.0 eV) [60] or PEDOT:PSS/P3HT:PCBM (2.2 eV) [61], they still have high potential as organic semiconductors. This is so because the value of their band gaps is lower than those for heptacoordinated tin(IV) complexes embedded in PEDOT:PSS (2.83–3.47 eV) [62,63,64]. The band gap values obtained for PEDOT:PSS-salophen derivative are lower than those for difluoroboron β-diketonate films in PEDOT:PSS (3.87 eV) [49]. This is remarkable, considering that salophen derivatives do not have metal atoms that promote charge transport through the films. Thus, their relatively low $E_{g}^{opt}$ values allow their use in optoelectronic devices, such as OLEDs and organic solar cells, which employ semiconductors with band gaps below 3.5 eV. The smaller the band gap of an organic semiconductor film, the greater the charge transport in it.

**Figure 9:**
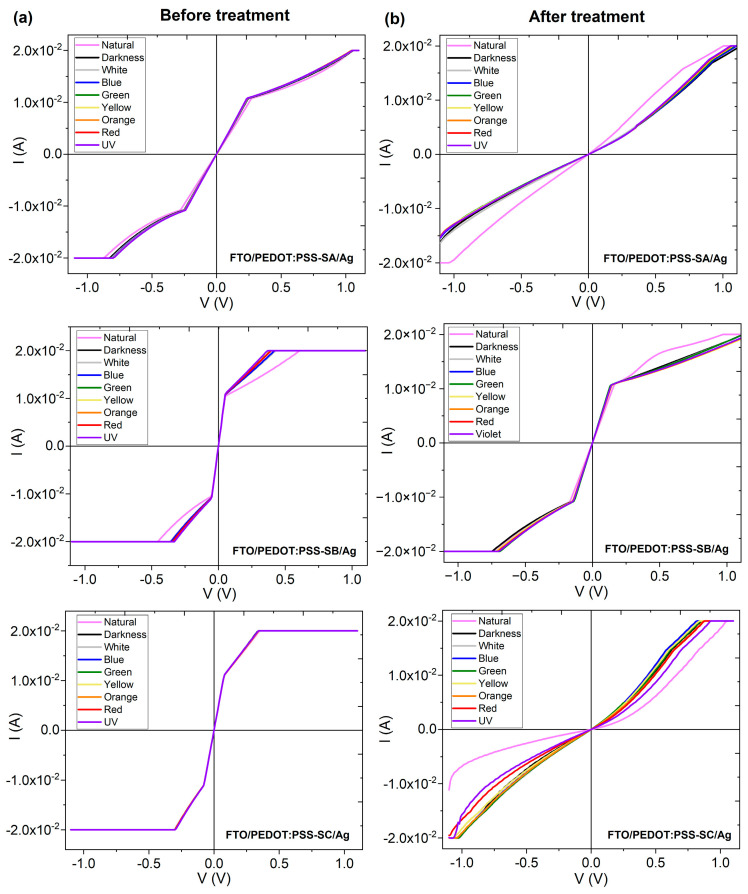
Current–voltage curves for glass/FTO/PEDOT:PSS-salophen/Ag devices (**a**) before and (**b**) after the IPA treatment.

In order to study the electrical behavior of the composite films, glass/FTO/PEDOT:PSS-salophen/Ag monolayer devices were fabricated, where the FTO acts as the anode, while the Ag acts as the cathode. Figure 9a,b show the behavior of the three devices before and after the IPA treatment, respectively. The PEDOT:PSS-salophen films do not show ambipolar behavior; so, the type of electrodes in the devices is decisive. The devices were irradiated with light of different wavelengths, and, before the treatment, there is no significant effect of this radiation on the amount of electric current transported. After the IPA treatment, the natural lighting conditions modify the transport of electrical charges. The interactions among the PEDOT:PSS chains in the linear conformation of the quinoid form are stronger than those of the benzoid form before the treatment, and electrical charges can be transported more freely under natural light conditions and without the need for any type of excitation. On the contrary, under light or dark conditions, there is no change in the transported current because, in this case, the radiation is filtered based on its wavelength, and apparently, it is insufficient to increase the electrical charges circulating in the device. Although the maximum current of 0.02 A is the same in all cases, changes in the electrical behavior of the devices are observed after the IPA treatment. 

Before the treatment, in the devices with SA and SC, the behavior is approximately ohmic at voltage intensities of V ≤ 0.4 V and corresponds to space charge-limited conduction (SCLC) at higher voltages. After treatment, the behavior changes to ohmic up to voltages of 0.9 V. In the device with SB, the behavior is ohmic for V ≤ 0.1 V, SCLC up to V ≤ 0.4 V, and with constant current for V > 0.4 V. After the treatment, the behavior remains ohmic for V ≤ 0.3 V and SCLC for higher voltages. From the above, it follows that the IPA treatment and, above all, the quinoid form of PEDOT:PSS is a determining factor in the electrical behavior of the devices. This does not translate into an increase in the transported electric current, as normally occurs, but rather, leads to a change in the type of electrical behavior. In the ohmic regime, the current flows freely as the voltage increases, whereas, in the SCLC regime, areas of high charge concentration are generated, which are released as the voltage increases. The benzoid form leads to a decrease in the SCLC and promote a freer charge flow, which facilitates the use of these films in optoelectronic devices, due to a higher inter-chain interaction between the conducting PEDOT chains. The PEDOT-rich domains create more conducting percolation pathways for the electric current, thereby significantly changing electric behavior [65]. On the other hand, when comparing the graphs in Figure 9 with each other, it can be observed that each of the devices has its own behavior that also depends on the type of salophen in each composite film. In the glass/FTO/PEDOT:PSS-SA/Ag device before post-treatment, the behavior is ohmic at low voltages and, at higher voltages, it changes to SCLC, while, in the treated device, the behavior becomes mainly ohmic. In the case of the glass/FTO/PEDOT:PSS-SB/Ag device, the behavior changes from ohmic to SCLC; however, in the untreated device, the current remains constant, while in the treated IPA device, the current increases. Finally, the glass/FTO/PEDOT:PSS-SC/Ag device is the one that changes the least in terms of electrical behavior. In both cases, the behavior begins ohmic, then changes to SCLC, and the transported current remains subsequently constant, although the transitions occur at different voltages. The change “ohmic behavior → SCLC → constant current” occurs because, at low voltages, charges flow freely along the composite films with ohmic behavior; voltage increases lead to charge saturation, most likely in the interface between the polymer and salophen in the composite films, and then the behavior changes to SCLC. Saturation eventually leads to a threshold, beyond which the electric current remains constant. These results suggest that PEDOT:PSS-salophen derivative films may be used in such organic electronics applications as organic diode-type devices.

To further characterize the PEDOT:PSS-salophen films and define possible uses in organic electronics, the external quantum efficiency (EQE) was measured before and after the IPA treatment. EQE refers to the number of photons emitted by the device, with respect to the number of injected charges. The measured EQE from 375 to 1150 nm had average maximum values of 0.19% and 0.086% for the devices without treatment and with treatment, respectively. Although there are differences between each of the three devices, the EQE values are in the same range and are also in the same order of magnitude as those obtained for composite films with PEDOT:PSS and inorganic dopants, such as MoO_3_ or MoO_3_-Ammonia [66,67]. The low EQE is attributed to the presence of salophen derivatives, which, despite having adequate optical and electrical properties, do not seem to have effective photovoltaic properties. This may also be the reason why, in the study of their electrical behavior, the amount of transported electric current did not change when the lighting and/or dark conditions of the devices were modified. On the other hand, the value of J_SC_, which represent the maximum current that flows through an organic solar cell when the voltage across it is zero, was also evaluated. J_SC_ provides information on the ability of the glass/FTO/PEDOT:PSS-salophen/Ag devices to capture and use the AM1.5 spectrum. The values obtained are between 1 and 1.6 mA/cm^2^; they change depending on the type of salophen present in the device and on whether this device is treated or not with IPA. The values obtained of J_SC_ are lower than those from the PEDOT:PSS composite films with widely tested dopants, such as graphene [68] or MoO_3_ [66]. However, the salophen used in this work are organic compounds that, to the best of our knowledge, have not been used in the fabrication of PEDOT:PSS composite films. While the EQE and J_SC_ values are rather low, they may be increased in the future by the addition of interfacial layers between the PEDOT:PSS-salophen film and the electrodes.

## 4. Conclusions

A series of three salophen derivatives were synthesized by a solvent-free mechanochemical ball milling methodology of easy work-up. These salophen were embedded in a PEDOT:PSS polymer matrix and by means of spin coating. Homogeneous films were obtained that were treated with isopropanol to produce the quinoid form of the polymer. The PEDOT:PSS-salophen composite films have a maximum transmittance of 97% for the PEDOT:PSS-SB film and a minimum of 80% for the rest of the films. The $E_{g}^{opt}$ obtained is 3.2 eV for the PEDOT:PSS-SA, 2.86 eV for the PEDOT:PSS-SB, and 2.97 eV for PEDOT:PSS-SC, which puts these films in the category of organic semiconductors. The devices made from the three composite films carry a maximum current of 20 mA and their electrical behavior changes with the increase in the voltage. This is due to the type of salophen that makes up the composite film, and to the salophen–polymer interface. Additionally, the maximum current densities that the devices can transport at zero voltage were evaluated, as well as their efficiencies in terms of the number of photons emitted and the number of injected charges. Although the values obtained are low, future work is expected to include injector and transport layers for holes and electrons, which should increase device charge transport.

## Data Availability

Data are contained within the article.
